# Can Pyorrhea Alveolaris Be Cured? If so, What Constitutes a Cure?

**Published:** 1911-01-15

**Authors:** Austin F. James

**Affiliations:** Chicago


### CAN PYORRHEA ALVEOLARIS BE CURED? IF SO, WHAT CONSTITUTES A CURE?

BY AUSTIN F. JAMES, D.D.S., CHICAGO.

In coming before you with this subject, it is not my idea to give something new, but rather to encourage those of you who have been doing conscientious work in the local treatment of pyorrhea and assure you that you are the dentists who are really doing something to control the ravages of the one disease that is causing the loss of more teeth than all other pathological mouth conditions put together. And while offering encouragement, I also wish to protest against this flood of so-called sure-cure drugs which are offered to us by circular and by sample. Most of you, I am sure, will support me in the opinion that these drugs are of no use whatever and only serve to lend false hope to those who have not been so fortunate as to work out a thorough systematic plan of surgical and prophylactic treatment for the cure of this disease.

Pyorrhea is cured by local treatment, and by local treatment only. I need not make this positive statement to many of you, for you, as well as I, know it is true, but I make it to try and influence those who have been carried away by the teachings of those theorists who have misled us for many years with the idea that pyorrhea is the result of systemic conditions, and that a cure cannot be obtained except in connection with constitutional treatment. Some of the most prominent men in our profession have advocated this theory and I might say all but proven it, except to find a specific cause or cure a single case of pyorrhea by constitutional treatment.

When we get down to the study of uncleanly mouth conditions we have the answer to the question, "What causes pyorrhea alveolaris?"

The heat and moisture of the mouth causing fermentation of food particles, and the formation of mucous placques and of acids which attack tooth surfaces causing superficial caries, and a chemical reaction on the salivary secretions resulting in ropy, thickened saliva which adds to the difficulty of cleansing the teeth, either by the friction of food during mastication, or by the use of the brush. Until the time comes when we have a general infected condition in the mouth, with caries upon the teeth, an accumulation of salivary deposit, and irritation and inflammation of the gingival margins, and finally the formation of calcareous deposits under the gum margins, which bring about a congested conditon in the soft tissues and the breaking down of the alveolar process and the formation of pockets along the side of the roots, and the destruction of septum in the interproximal spaces.

Following this stage we have the serumal deposit forming on the roots of the teeth, and it is this serumal deposit that has caused the belief that pyorrhea comes from constitutional conditions. But we never find this serumal deposit until we have had an acutely congested condition of the soft tissues and the breaking down or absorption of the alveolar process, and we do not have the pus formation until there has been absorption of process and a pocket that cannot be cleansed, which finally becomes infected from the general septic condition prevailing, and it is not pyorrhea until this stage has been reached.

In the treatment of pyorrhea my first step is to thoroughly wash out all pockets and surfaces in and about the necks of the teeth with warm carbolized water, strongly blended with peppermint water. Then begin instrumentation, following out the definite plan of working only upon the number of teeth that can be finished at one sitting, and not leaving those teeth until I am sure all deposits have been removed and all roughened surfaces of enamel margins made perfectly smooth. Following instrumentation I dry the tissues about the teeth worked upon by packing well with cotton rolls and carrying phenol sulphonic acid into the pockets on a gold broach, the end of which has been looped or bent upon itself in order to carry the acid more readily. This acid can be used freely in the pockets so long as you keep saliva away from it and by wiping away any excess before having the patient rinse the mouth with water. This drug is used for the purpose of cauterizing the surfaces in order to prevent absorption of any infectious matter until the process of healing begins, when there is little danger from this source. From this time on I carry out what I call the proving up treatment, which is nothing more than prophylaxis, or the perfect polishing of all surfaces of the teeth with wood points shaped in a variety of ways to accommodate the different positions in the mouth, extending the polishing process up against the gums and under the free margins wherever possible, with the idea of getting the tooth surface so polished that the patient can rinse them cleaner than they have ever been able to brush them before. In this manner we get rid of all fermentation and further irritation from uncleanly conditions. In the meantime I have the patient follow out the plan of massaging the gums for three minutes twice a day, using ivory soap on the brush, not for cleansing purposes, but to take away the harshness of the brush.

I tell the patient that this is the one way of finding out whether I have been successful in removing all deposits. If so, the gums will soon harden under the massaging; if not, the gums will show a point of irritation wherever the deposit has been missed, and I can make a second effort at that particular point. Putting this plan before the patient usually stimulates him to greater effort, as he feels that it is proving up my work and is naturally anxious to make sure.

I have avoided saying anything about the kind of instruments used in removing deposits, because I have a hesitancy about advertising manufacturers in presenting a paper before your society; but I think the instruments are the most important thing to be considered outside of thoroughness in the work, and this work cannot be done without the proper form of instrument, which should be a set of sufficient number, shape and angle to accommodate every size tooth and position in the mouth.

Such a set has been devised by C. M. Carr, and is composed of one hundred and fifty instruments, constructed on the principle of a plane; the instrument using the tooth as a fulcrum by resting on the tooth anterior to the blade of the instrument, rotating it upon the tooth with a slight backward, or reverse movement, usual to the pull instruments so commonly used, thus making it possible to actually plane the surface of the root with a slight delicate stroke which enables one to detect the smallest deposit and know when it has been removed, so acute becomes the sense of touch after mastering the technique of the instrument.

We now come to the second question of my subject as to what constitutes a cure of pyorrhea alveolaris.

The cure of pyorrhea does not mean the restoring either of lost bone or of the gum tissues, but rather the obliteration of all pockets by forcing the gum tissue to recede to a point where there is healthy bone to support it. Nor does it mean that all loose teeth can be made tight without the aid of a splint or a permanent retaining appliance.

There are times when we apparently get bony granulation and the seeming filling up of pockets by the formation of healthy tissue but I have never found a definite way of bringing this about, though it seems to come in some cases following the surgical treatment. It has been my observation, however, that it usually happens where no teeth have been lost; and it has been possible to restore normal contact points and correct occlusions. Though this is not to be depended on at any time.

The obliteration of pockets follows the successful surgical treatment and keeping the mouth rid of fermentation by prophylaxis, which means thorough and continuous polishing of all tooth surfaces until they can be kept clean by the use of the brush in the patient's hand.

The number of sittings required to obtain prophylaxis varies in each mouth. One has to keep up these repeated treatments until the result has been obtained The patient will come back to you after a period of one to three months without stains or mucous placques on the teeth the gums showing a hard, glistening surface, with no points of irritation, and no sensitiveness of the necks of the teeth.

The splinting of loose teeth in molars and bicuspids is accomplished by getting separation and building exaggerated contour inlays, attaching at contact points by solder, and cementing into place.

In the anterior teeth I use a lingual backing for each tooth, with small pins. Taking an impression with all in place remove solder and cement into place as a permanent retention.

In cases where there is so much absorption and recession that we have an elongated and unsightly condition I cut off these teeth and make attached crowns to improve the appearance and serve as a permanent retainer.

As to medication, I wish to add, not as an after thought but because I use it only where I find peculiar root formation, and merging bifurcations which prevent successful instrumentation, that Head's Tartar Solvent is an invaluable aid, and that its action upon the teeth and soft tissues need not be feared, if Dr. Head's directions are followed and force is not used when flooding the pockets with the drug.—*Western Journal.*

